# Molecular modeling and molecular dynamics simulations based structural analysis of the SG2NA protein variants

**DOI:** 10.1186/1756-0500-7-446

**Published:** 2014-07-11

**Authors:** Sangeeta Soni, Chetna Tyagi, Abhinav Grover, Shyamal K Goswami

**Affiliations:** 1 School of Life Sciences, Jawaharlal Nehru University, New Delhi 110067, India; 2 School of Biotechnology, Jawaharlal Nehru University, New Delhi 110067, India

**Keywords:** SG2NA, Molecular modeling, Molecular dynamics simulations, Disorder prediction, Striatin

## Abstract

**Background:**

SG2NA is a member of the striatin sub-family of WD-40 repeat proteins. Striatin family members have been associated with diverse physiological functions. SG2NA has also been shown to have roles in cell cycle progression, signal transduction etc. They have been known to interact with a number of proteins including Caveolin and Calmodulin and also propagate the formation of a multimeric protein unit called striatin-interacting phosphatase and kinase. As a pre-requisite for such interaction ability, these proteins are known to be unstable and primarily disordered in their arrangement. Earlier we had identified that it has multiple isoforms (namely 35, 78, 87 kDa based on its molecular weight) which are generated by alternative splicing. However, detailed structural information of SG2NA is still eluding the researchers.

**Results:**

This study was aimed towards three-dimensional molecular modeling and characterization of SG2NA protein and its isoforms. One structure out of five was selected for each variant having the least value for C score. Out of these, m35 kDa with a C score value of −3.21was the most poorly determined structure in comparison to m78 kDa and m87 kDa variants with C scores of −1.16 and −1.97 respectively. Further evaluation resulted in about 61.6% residues of m35 kDa, 76.6% residues of m78 kDa and 72.1% residues of m87 kDa falling in the favorable regions of Ramchandran Plot. Molecular dynamics simulations were also carried out to obtain biologically relevant structural models and compared with previous atomic coordinates. N-terminal region of all variants was found to be highly disordered.

**Conclusion:**

This study provides first-hand detailed information to understand the structural conformation of SG2NA protein variants (m35 kDa, m78 kDa and m87 kDa). The WD-40 repeat domain was found to constitute antiparallel strands of β-sheets arranged circularly. This study elucidates the crucial structural features of SG2NA proteins which are involved in various protein-protein interactions and also reveals the extent of disorder present in the SG2NA structure crucial for excessive interaction and multimeric protein complexes. The study also potentiates the role of computational approaches for preliminary examination of unknown proteins in the absence of experimental information.

## Background

SG2NA (Striatin-3) is a WD-40 repeat protein, originally identified as a nuclear auto-antigen with augmented expression during S to G2 phase of cell cycle [1]. Subsequent studies established its structural and functional relatedness to striatin and zinedin, thereby constituting a three member sub-family of WD-40 superfamily [2-4]. In addition to WD-40 repeats at the carboxylic terminal, they also contain a caveolin binding motif, a coiled-coiled structure and a calmodulin binding domain located in the same order at the amino terminus [3]. As expected from these domain organizations, Striatin family members have been shown to interact with a diverse group of proteins including the regulatory subunit (C subunit) of serine/threonine protein phosphatase PP2A [5], caveolin [6], tumor suppressor protein APC [7] and estrogen receptor [8]. Recently, a large multiprotein complex named Striatin-interacting phosphatase and kinase (STRIPAK) containing Mob3, STRIP1, STRIP2, the cerebral cavernous malformation 3 (CCM3) protein, and Ste20 kinase has been described [9]. In agreement with the existence of diverse interacting partners, Striatin family members have been associated with functions as diverse as non genomic estrogen signalling [8], cytokinesis [9], spinogenesis [10], Golgi polarization [11] and vesicular trafficking [12]. Members of the striatin family are conserved in metazoan evolution but are absent in plants and prokaryotes. Also, SG2NA is the first among the three to evolve from prokaryotes [13]. Noticeably, *Drosophila* has only one Striatin homologue i.e., CKA; which acts as a platform for organizing the components of JNK signaling and the transcription factor AP-1, suggesting similar but distinct functions of the three members in higher metazoans [3,14].

Although SG2NA has been shown to have roles in cell cycle progression, non-genomic estrogen signaling and signal transduction; the structural basis of such diverse functions is not known. Earlier we had shown that it has multiple isoforms generated by alternative splicing [4]. Variants of SG2NA localize in cytosol, mitochondria, plasma membrane, lysosomes and nucleus (unpublished results). It is thus likely that variants of SG2NA perform multiple functions in a context specific manner. Further, due to the presence of WD-40 repeats, it is anticipated that SG2NAs have scaffolding function, but the interactomes are not known.

Since most of the functional properties of the proteins intricately depend upon their structures, the requirement of the 3D structures is indispensable for understanding the biological functions. Three of the mouse SG2NA variants (35 kDa, 78 kDa, and 87 kDa) have been cloned and purified. They consist of 301, 712 and 796 amino acid residues respectively. Since among homologous proteins, structure is more conserved than the sequences, the three-dimensional (3D) structure prediction of SG2NA variants can help us in the predicting their functions. The crystallographic structure of SG2NA has not been reported yet and its active site residues have also not been identified. In the present study we carried out a molecular modeling study of SG2NA proteins using iterative threading assembly refinement (I-TASSER) to obtain their 3D structures. These structures were further energy minimized and corrected by molecular dynamics (MD) simulations to arrive at a better model. The simulations of SG2NA and its isoforms were performed for a long time duration of 10 ns and the stabilized structures so obtained were further analyzed.

## Methods

### Retrieval of sequence data for SG2NA protein variants

The amino acid sequences of SG2NA protein variants (35 kDa, 78 kDa, and 87 kDa) were retrieved from UNIPROT (IDs: B3STQ1 [4], B2RQS1 [15] and Q9ERG2 [2] respectively). These SG2NA sequences were used to screen similar sequences with known 3D structures against Protein Data Bank (PDB) using NCBI’s BLAST program [16].

### Primary structural analysis of SG2NA protein variants (35 kDa, 78 kDa and 87 kDa)

Expasy’s Prot Param server [17] was used to study the physiochemical characters of SG2NA protein variants such as theoretical isoelectric point (pI), molecular weight, molecular formula, total number of positive and negative residues, instability index [18], extinction coefficient [19], aliphatic index [20] and grand average hydropathy (GRAVY) [21,22]. CYS_REC tool has been applied for the anticipation of disulphide bond (S-S) pattern. The instability index provides the estimate of a protein’s stability in vitro and is based on the difference in occurrence of certain dipeptides in the unstable and stable proteins and calculated by assigning weights of unstability to 400 different dipeptides (DIWV) and using the equation [Eq 1]

(1) $$\mathrm{Instability}\;\mathrm{Index}\;=\;\left(10/L\right)*\mathrm{Sum}\;\mathrm{DIWV}\left(x\left[i\right]x\left[i\;+\;1\right]\right)$$

where *i* = 1, L is the length of the sequence and DIWV(x[*i*] x[*i* + 1]) stands for the instability weight value for the dipeptide starting from positon *i*. The extinction coefficient predicts the amount of light a protein can absorb at a certain wavelength. It can be calculated for any protein given the molar extinction coefficients of Trp, Tyr and Cys are provided, using the following equation [Eq 2].

(2) $$\begin{array}{l} E\left(\mathrm{Prot}\right)\;=\;\mathrm{Numb}\left(\mathrm{Tyr}\right)*\mathrm{Ext}\left(\mathrm{Tyr}\right)\\ \;+\;\mathrm{Numb}\left(\mathrm{Trp}\right)*\mathrm{Ext}\left(\mathrm{Trp}\right)\\ \;+\mathrm{Numb}\left(\mathrm{Cys}\right)*\mathrm{Ext}\left(\mathrm{Cys}\right) \end{array}$$

where (for proteins in water measured at 280 nm): Ext(Tyr) = 1490; Ext(Trp) = 5500; Ext(Cys) =125. The aliphatic index can be regarded as a positive factor in increased thermostability of globular proteins by measuring the relative volume occupied by aliphatic side chains (alanine, valine, leucine and isoleucine). It is calculated using the formula given in Eq 3.

(3) $$\begin{array}{l} \mathrm{Aliphatic}\;\mathrm{Index}\;=\;X\left(\mathrm{Ala}\right)\;+\;a*X\left(\mathrm{Val}\right)\\ \;+\;b\;*\left(X\left(\mathrm{Ile}\right)\;+\;X\left(\mathrm{Leu}\right)\right) \end{array}$$

where X(Ala), X(Val), X(Ile), and X(Leu) are mole percent (100 X mole fraction) of alanine, valine, isoleucine, and leucine. The coefficients *a* and *b* are the relative volume of valine side chain (*a* = 2.9) and of Leu/Ile side chains (*b* = 3.9) to the side chain of alanine.

The GRAVY score is the ratio of the sum of hydropathy values of all amino acids and the number of residues in the sequence.

### Secondary structural analysis of SG2NA protein variants

Secondary structural analysis was done using PSIPRED view web server [23]. It is a new and highly accurate secondary structure prediction method. It was employed to enumerate the secondary structural features of SG2NA proteins Two feed-forward neural networks integrated by PSIPRED, perform an analysis on output received from PSI-BLAST (position specific Iterated BLAST). A putative secondary structure is obtained with respect to each residue associated with a confidence value for the prediction.

### Ab Initio modeling, Structure refinement and identification of functional site

Pertaining to lack of suitable structural template for SG2NA and its isoform, we resorted to *ab initio* modeling of SG2NA using I-TASSER [24,25], a web based, full chain protein prediction server. I-TASSER follows a comprehensive protocol for protein modeling. It first scans a representative PDB structure library to search for possible folds by threading the target sequence using Profile-profile threading alignment (PPA). The threading aligned regions are excised and threading unaligned regions are modeled using *ab initio* modeling before reassembling the complete model together. The knowledge of a protein’s conformational space is crucial and to search that it uses replica exchange Monte-Carlo simulations. Models having conformational similarities are clustered by SPICKER. By averaging the coordinates of structures in a cluster, a cluster centroid is obtained. To rule out steric clashes and refine the models further, fragment assembly simulation is done starting from the cluster centroid obtained from the previous simulation. After retrieval of spatial constraints from these clusters, TM-align is used to search for PDB structures to guide second simulation. After clustering the structure decoys, the lowest energy structure in each cluster is chosen. Simulations are run for full chain as well as the domains. Finally, the complete model is obtained by docking the model of domains together by Metropolis Monte Carlo simulation. This docked complex is compared to the I-TASSER full chain model and arrives at the closest one with least steric clashes.

The C score is defined by

$$C\;\mathrm{score}=\ln\;(\;\frac{M}{M_{\mathrm{tot}}}.\;\frac{1}{\mathrm{RMSD}}.\;\frac{\prod_{i=1}^{4}Z\left(i\right)}{\prod_{i=1}^{4}\mathrm{Zo}\left(i\right)})$$

where M is the multiplicity of structures in the SPICKER cluster; M_tot_ is the total number of the I-TASSER structure decoys used in the clustering; RMSD is the average RMSD of the decoys to the cluster centroid; Z(i) is the highest Z score (the energy to mean in the unit of standard deviation) of the templates by the i^th^ PPA threading program and Z_0_(i) is a program-specified Z-score cutoff for distinguishing between good and bad templates, i.e. Z_0_(1) = 7.0, Z_0_(2) = 8.5, Z_0_(3) = 8.0, Z_0_(4) = 10.5.

The TM score is defined to assess the topological similarity of two proteins

Mscore=1L∑i=1L11+di2d02

where *d*_*i*_ is the distance of the *i*^th^ pair of residues between two structures after an optimal superposition, and *L* is the protein length. TM score lies in between 0 to 1 with higher values indicating better models. Statistically, a TM-score ≤ 0.17 corresponds to a similarity between two randomly selected structures from the PDB library while a TM-score > 0.5 corresponds approximately to two structures of the similar topology. Structure based protein functional site of SG2NA and of three isoforms were predicted by Q-site Finder [26].

### Structure Validation of the models obtained by I-TASSER

I-TASSER server provided five structures for each of the protein. Then PSVS web server was used to assess the qualities of the models. The models were evaluated by Verifiy3D, Prosall, PROCHECK [27] and Molprobity programs to check the correctness of the overall fold/structure, errors over localized regions and stereochemical parameters such as bond lengths and angles. Structural validation of target proteins model were done to determine stereochemical aspects along with main chain and side chain parameters with comprehensive analysis. This shows the various residues of SG2NA falling under allowed, favoured and disallowed regions of a Ramchandran plot obtained from PROCHECK and Richardson Lab’s MolProbity [27]. The post-simulated structures were also assessed for their quality using the same server.

### Molecular Dynamics simulations of SG2NA variants (35, 78 and 87)

The AMBER v.10 package [28] was used to prepare the proteins for the Molecular Dynamics (MD) simulations. The coordinates of protein atoms were optimized in the protein preparation wizard of Schrodinger’s Maestro suite where hydrogens were added; water molecules and UDP were removed, and the complex structure was minimized using the OPLS2001 force field. The 1-μm simulation used the CHARM27 forcefield [29], and the simple point charge model for water. The CHARM27 forcefield was applied to the system using the VIPARR utility. The default Desmond relaxation was performed before simulation, and molecular dynamics were run at constant temperature (300 K) and pressure (1 bar). The simulation was performed by using the program Desmond, compiled by SB Grid on an optimized 64-node Linux-based InfiniBand cluster.

### Comparative split-domain modeling using QUARK and MUSTER algorithms

The SG2NA isoforms m78 and m87 were split into three domains namely, caveolin-binding and putative coiled-coil (res. 1–140), calmodulin binding domain (res. 141–300) and the WD 40 repeat domain (301–712 residues in m78 and 301–797 residues in case of m87 respectively) owing to long amino acid sequences. As the first two domains were small in size, they were modeled using ab initio server QUARK [30] and the last one was submitted to template based server MUSTER [31]. QUARK is a template-free prediction tool which accepts sequences of less than 200 residues. It splits the sequences into smaller fragments up to of 1–20 residues and obtains a number of fragment structures from unrelated experiments for each position. Using replica-exchange Monte Carlo simulations the fragments are assembled based on knowledge based force fields to yield complete structural models. On the other hand, MUSTER is template-based modeling software. It stands for MUlti-Source ThreadER and is an extension to the simple threading algorithm named, PPA. The algorithm uses dynamic programming methods and includes single-body features like (1) sequence profiles (2) secondary structure prediction (3) depth-dependent structure profiles (4) solvent accessibility (5) backbone dihedral torsion angles and (6) hydrophobic scoring matrix. After selection of appropriate templates the models are built using the inbuilt Modeller algorithm.

### Disorder prediction of SG2NA proteins

The disorder prediction for SG2NA proteins was done using a web-based server named IUPred [32] which is based on the assumption that relatively stable proteins can form inter-residue contacts that stabilize the entropy loss during folding while the intrinsically disordered proteins (IDPs) of which Striatin is also a part, have certain sequence regions that are unable to form sufficient inter-contacts. In this method, the pairwise interaction energies of a few selected globular proteins can be estimated using a quadratic expression set for each residue in terms of whether it contributes to the ordered or disordered regions. This contribution depends upon its own chemical composition as well as its sequential interaction partners as a whole. It resulted in a disorder prediction graph along with the disorder probability for each residue position.

## Results & discussion

SG2NAs belong to the Striatin family constituted of two other members i.e., Striatin and Zinedin. Although the expression of mammalian Striatin is largely restricted to the central nervous system (CNS), that of SG2NA is ubiquitous [6,13]. All three members share a number of protein-protein interaction domains like WD-repeat (except 35 kDa SG2NA), Ca^2+^-calmodulin binding and the putative coiled-coil structure, suggesting that they are involved in Ca^2+^ dependent signal transduction. Thus, the knowledge of their structure is imperative for a better understanding of their functions. The three variants of the SG2NA viz., 35 kDa, 78 kDa and 87 kDa differ in their size and sequences. In agreement, structures with different topology were observed.

### Primary structural analysis of SG2NA protein

The primary structural features of SG2NAs (35 kDa, 78 kDa, and 87 kDa) are described and compared in Table 1. The calculated isoelectric points (pI) for all three isoforms are 5.90, 5.06 and 5.12 respectively, suggesting the presence of more negatively charged residues. Amidst the three isoforms under study, the 87 kDa possesses higher extinction coefficient, indicating the presence of Cys, Trp and Tyr in abundance. Higher number of these residues aid to the quantitative study of protein-protein and protein-ligand interactions in solution. The disulphide (S-S) bonding pattern depicts three cysteine residues in 78 kDa variant at positions 544, 546 and 670 and two cysteine residues in 87 kDa variant at positions 556 and 613. The 35 kDa variant appears to have no disulphide bond. As the presence of a disulphide bond increases the enthalpy of the folded state by stabilizing local interactions, these results suggest that SG2NA has high enthalpy at the folded state. Although, at a wide range of temperatures SG2NAs were found to be stable (due to the higher aliphatic index [AI]), an instability index of more than 40 suggests instability and thus predicts that the variants of SG2NA are thermally unstable [33]. The value of aliphatic index for SG2NAs (35 kDa, 78 kDa, and 87 kDa) were 69.44, 78.40 and 77.60 respectively while that of instability index were 60.06, 46.0 and 45.15. GRAVY (Grand average hydropathy) values of SG2NAs were −0.869, −0.470 and −0.532, suggesting a hydrophilicity pattern with better interaction with water. Also, the GRAVY value of 35 kDa (−0.869) was lowest among all three variants, indicating a better solubility as compared to 78 and 87 kDas.

**Table 1:** Physico-chemical properties of three SG2NA isoforms

**Sequence variant**	**35 kDa**	**78 kDa**	**87 kDa**
**Accession number (from Uniprot)**	B3STQ1	B2RQS1	Q9ERG2
**Molecular formula**	C_1480_H_2325_N_429_O_454_S_6_	C_3426_H_5339_N_947_O_1081_S_20_	C_3825_H_5995_N_1069_O_1216_S_23_
**Molecular Weight**	33584.7	77731.9	87150.4
**p.I.**	5.90	5.06	5.12
**-R (negative residue)**	44	100	117
**+R (positive residue)**	39	66	81
**Extinction Coefficient**	29910	89185	96300
**Instability Index**	60.06	46.00	45.19
**Aliphatic Index**	69.44	78.40	77.60
**GRAVY**	−0.869	−0.470	−0.532
**S-S Bond**	None	544, 546, 670	556, 613

### Secondary structure prediction of SG2NA proteins

Currently, most of the algorithms for the anticipation of secondary structure of a protein are based on machine learning techniques. Among the available methods to date, PSIPRED-view achieved highest level of accuracy, an average Q3 score of 76.5% [34]. It was used for the prediction and analysis of secondary structure of SG2NA proteins and is shown in Table 2. Only α helices were found in the predicted structure of 35 kDa SG2NA, It reveals the unfavored structural property in non-polar solvent. The amount of alpha helix, beta sheets, and coils in 78 SG2NA are 13.5%, 23.7% and 62.78% respectively while that of in 87 kDa are 13.94%, 20.22%, and 65.82% respectively. These results can help in experimental verification of a predicted folding motif as it may be gained by measurements of protein secondary structural elements of which the motif is composed. Another insight into the secondary structure of these isoforms was provided by the PSVS validation suite and has been summarized in Table 2 along with PSI-PRED results to compare the accuracy of the servers and reach a consensus. According to PSVS, 35 kDa SG2NA is constituted of only α-helices which amount to 26.24% of the complete protein. In case of the 78 kDa isoform, amount of β-sheets exceeded α-helices standing at 25.28% and 10.81% respectively. For the 87 kDa isoform, α-helices take up 7.66% and β-sheets take up 28.39% of the complete structure.

**Table 2:** Secondary structure analysis of the three SG2NA isoforms using PSI-PRED and PSVS validation suite

**Variant (number of residues in%)**	**PSI-PRED**			**PSVS**		
	**α-helix**	**β sheets**	**Coils**	**α-helix**	**β-sheets**	**Coils**
**m35kDa**	40.1	0	59.9	26.24	0	73.76
**m78kDa**	13.5	23.7	62.78	10.81	25.28	63.91
**m87kDa**	13.94	20.22	65.82	7.66	28.39	63.95

### Prediction of three dimensional (3D) structures of 35 kDa, 78 kDa and 87 kDa SG2NA

**Figure 1:**
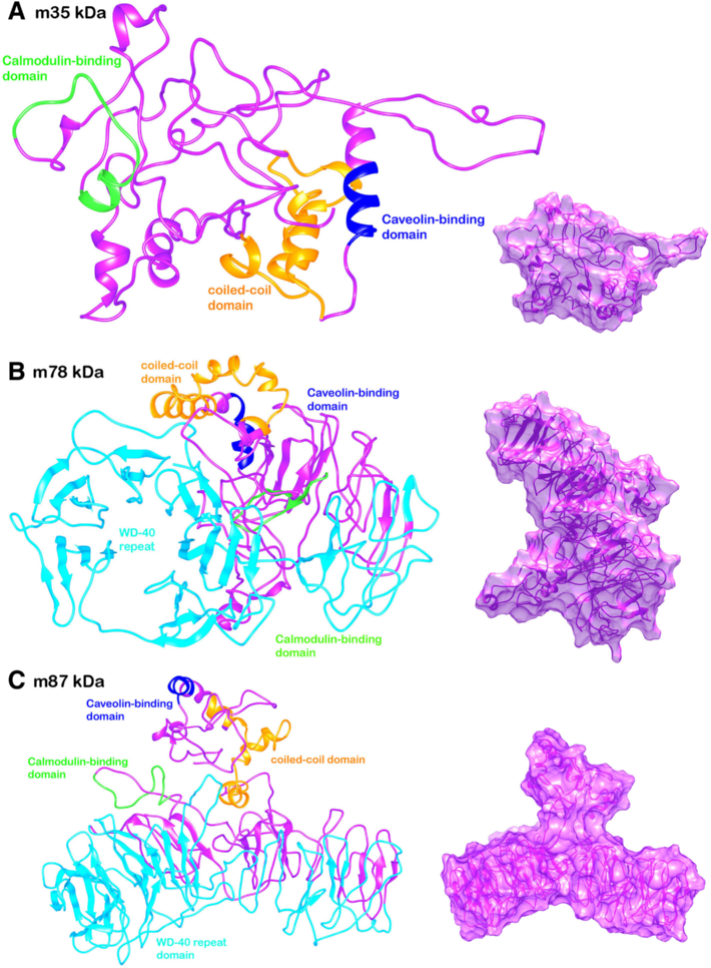
**Diagram depicting 3D topological structure of the SG2NA protein variants (A) 35kda (B) 78 kDa (C) 87 kDa.** The Caveolin-binding domain in blue (res. 71–79), putative coiled-coil domain in orange (res. 86–132), Calmodulin-binding domain in green (res. 166–183) and WD-40 repeat domain in cyan (res. 353–753) has been shown in both backbone and surface topology styles.

Three dimensional structure of SG2NA proteins were predicted and compared. The comparative protein structure analysis for SG2NA variants is still untouched and unavailable. The tertiary structure prediction was performed by I-TASSER server using the best aligned template obtained by searching against Protein Data Bank database. The search for optimal templates is inbuilt in the I-TASSER algorithm. Templates that were chosen are listed: 1wp1A, the OprM lipoprotein of *Pseudomonas aeruginosa* for 35 kDa and 2ymuA, WD-40 repeat protein of *Nostoc punctiforme* for 78 kDa and 87 kDa. The template was selected to model and analyze 3D structure because a high level of sequence identity should guarantee a more accurate alignment between the target sequence and template whose structure is known. The final template chosen is used to model the initial structure. This final template is chosen after aligning the query sequence with a number of other protein sequences whose structures are already known. Out of five generated models of the target sequence, the best ones have been chosen owing to the criteria of good alignment with the chosen template and are measured by C-Score, TM score and RMSD values as provided in Table 3. The developed 3D model of SG2NA proteins were deposited to the PMDB database. The diagram showing the post simulated topological structures of the three variants has been provided in Figure 1.

**Table 3:** Analysis of three dimensional structures of SG2NA protein variants obtained by I-Tasser based on quality parameters

**Variants**	**C score**	**TM score**	**RMSD**	**No. of decoys**	**Cluster density**
**m35kDa**	−3.21	0.36 ± 0.12	14.0 ± 3.9	940	0.0498
**m78kDa**	−1.16	0.57 ± 0.15	10.9 ± 4.6	381	0.0943
**m87kDa**	−1.97	0.48 ± 0.15	13.2 ± 4.1	292	0.0404

The C score refers to the confidence in the quality of the predicted structure and is based on threading template alignments and convergence parameters involved in simulation. It lies between −5 to 2, where a higher C score signifies absolute quality of the model and vice versa. The isoform 35 kDa with a C score of −3.21 is a poor model in comparison to the 78 kDa and 87 kDa models having a score of −1.16 and −1.97 respectively. To measure the structural similarity between two structures consequential in determining the quality of the predicted model, TM score and RMSD are calculated. But, owing to the error-prone state of the RMSD calculations, we rely on the TM score in which the smaller distances between structures are weighted stronger than the larger distances thus making it free of local errors. A TM value of more than 0.5 ensures a model of correct topology and a value of less than 0.17 refers to random similarity and hence should be discarded. The isoform 35 kDa with a TM score of 0.36 ± 0.12 is a poor model in comparison to the 78 kDa and 87 kDa models having a score of 0.57 ± 0.0.15 and 0.48 ± 0.15 respectively.

As discussed before, I-TASSER works by excising continuous fragments from the template alignments and reassembles those using Monte Carlo simulations. These low temperature replica structures also called decoys are clustered using SPICKER. The number of such decoys in a unit space of a SPICKER cluster is known as the cluster density, a high value representing how often a structure occurs in the simulation trajectory, signifies good quality of a model. The total number of structure decoys employed to model a structure has been provided in Table 3. The isoform 78 kDa with the highest value of cluster density at 0.0943 amongst its counterparts, 35 kDa and 87 kDa with cluster density of 0.0498 and 0.0404 respectively, signifies a model of better quality.

### Structure validation of the modeled SG2NA isoforms using PSVS

The quality of the predicted structures of SG2NA were further assessed and confirmed by Verifiy3-D, Prosall, PROCHECK, and Molprobity which are a part of the PSVS server. The scores (from −1 to +1) were added and plotted for individual residues. The residues falling in the area where the orange line crosses 0.0 have low prediction accuracy and less stable conformation whereas, most of the residues fall above 0.15-0.4 so we can say that the model is of good quality. The stereochemical quality and accuracy of the predicted model of SG2NA were evaluated after the refinement process using Ramachandran Map calculation with the PROCHECK program. The Ramachandran plot has been shown a tight clustering of phi ~ −50 and psi ~ −50. This plot is a way to visualize dihedral angles φ against ψ of amino acid residues in protein structure. It shows the possible conformations of φ and ψ angles for a polypeptide. In Ramachandran plot, the white areas correspond to conformations where atoms in the polypeptide come closer than the sum of their Vander Waals radii. These regions are sterically disallowed for all amino acids except glycine, which is unique in that it lacks a side chain. The red regions correspond to the allowed regions namely the alpha-helical and beta-sheet conformations where there are no steric clashes. The yellow areas show the partially allowed regions of left handed helix wherein the atoms are allowed to come a little closer together. Glycine is restrained by triangles and other residues are represented by squares.

**Figure 2:**
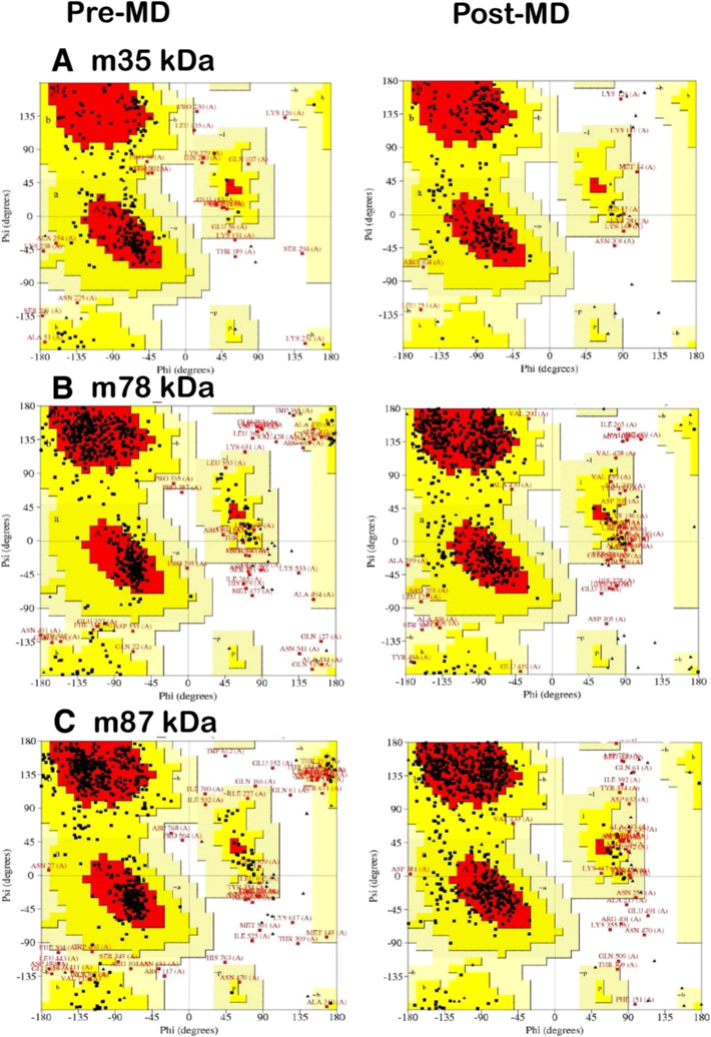
**Comparative diagram depicting Ramchandran Plot analysis of SG2NA protein variants before and after molecular dynamic simulations (A) 35kda (B) 78 kDa (C) 87 kDa.** Ramachandran plots show the phi (φ)-psi (ψ) torsion angles for all the SG2NA aminoacid residues in the structure. Glycine and proline residues are shown as triangles (▲) and are not restricted to the regions of plots. The plots were generated in PROCHECK.

The Ramchandran plot analysis obtained by PROCHECK and Richardson Lab’s MolProbity have been summarized in Table 4 and the plots are provided in Figure 2A. The residues falling in most favored regions of the Ramchandran plot obtained by PROCHECK amounted to 61.6%, 76.6% and 72.1% for m35kDa, m78kDa and m87kDa respectively while for the plot obtained by MolProbity it amounted to 69.9%, 80.7% and 78.5% respectively. Similarly, residues falling in disallowed regions amounted to 2.5%, 2.7% and 2.4% in PROCHECK while it stood at 9.7%, 7.6% and 8.7% obtained by MolProbity for the three SG2NA variants respectively. This provides an insight into the correctness of the modeled structures, m78kDa being the best modeled structure both in terms of PROCHECK and MolProbity. A low number of residues falling in the favored regions signify errors in the modeled structure of m35kDa protein.

**Table 4:** Ramchandran analysis for the validation of SG2NA proteins modeled using Procheck and MolProbity tools integrated in the PSVS servers before and after molecular dynamic simulations

**Residues**			**Most favored (%)**	**Allowed regions (%)**	**Generously allowed (%)**	**Disallowed (%)**
**m35kDa**	Pre-MD	Procheck	61.6	29.8	6.2	2.5
		MolProbity	69.9	20.4	-	9.7
	Post-MD	Procheck	74.0	22.3	2.9	0.8
		MolProbity	86.0	12.7	-	1.3
**m78kDa**	Pre-MD	Procheck	76.6	16.9	3.8	2.7
		MolProbity	80.7	11.7	-	7.6
	Post-MD	Procheck	73.3	20.3	4.5	2.0
		MolProbity	84.1	12.7	-	3.2
**m87kDa**	Pre-MD	Procheck	72.1	20.5	5.0	2.4
		MolProbity	78.5	12.8	-	8.7
	Post-MD	Procheck	74.3	21.3	2.8	1.6
		MolProbity	83.5	12.7	-	3.8

### Molecular dynamics simulations of SG2NA protein structures

**Figure 3:**
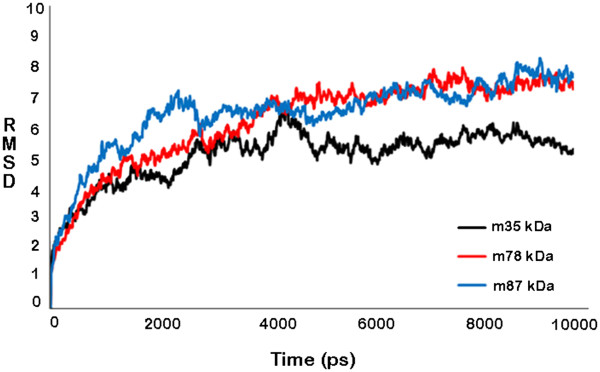
A comparative RMSD graph showing simulation trajectory acquired by the three variants where black curve refers to 35 kDa, red refers to 78 kDa and blue refers to 87 kDa isoforms.

**Figure 4:**
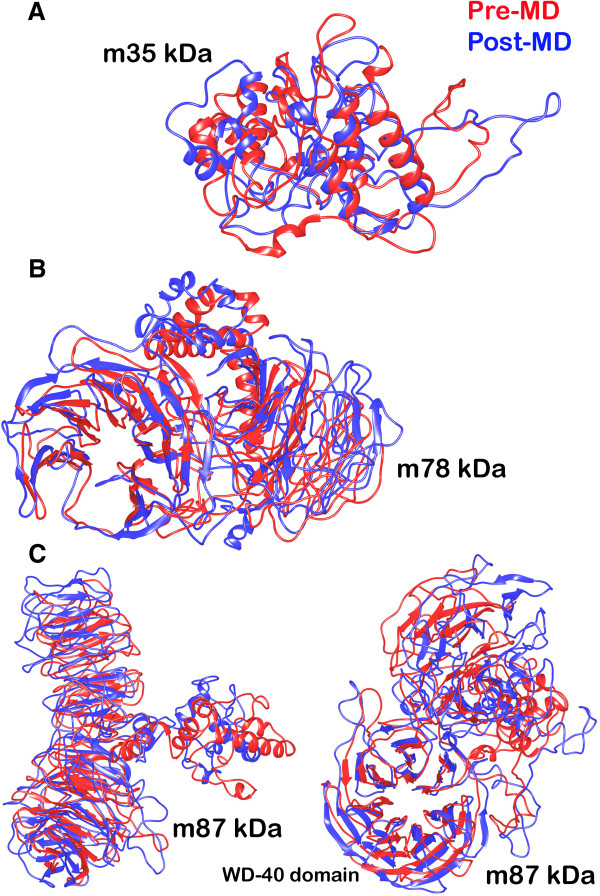
Comparative topological structure depiction of the three SG2NA variants (A) 35kda (B) 78 kDa (C) 87 kDa (in two configurations), obtained before (shown in red) and after molecular dynamics simulations (shown in blue).

Molecular dynamics simulations were performed on Desmond utility provided in the Schrodinger’s Maestro suite to ascertain the absolute quality of the models obtained to refine them by subjecting to 10000 picosecond (ps) long simulations. The final structures were obtained by taking the average of structures pertaining to that part of the trajectory where it becomes stable. A comparative trajectory graph has been provided in Figure 3 for the three SG2NA variants: m35kda, m78kDa and m87kDa. These post-simulated structures were again subjected to validation by the PSVS suite and the results have been summarized in Table 4 for comparison with the pre-simulated structures. As can be gathered from the results, the number of residues falling under most favored regions and allowed regions in the Ramchandran plot (both from PROCHECK and MolProbity) have significantly increased for all the three variants especially for m35kDa. These results are obtained due to the removal of unfavorable contacts from the protein model during MD simulation. This may, however, affect the global quality of protein; a limitation that is faced due to unavailability of more accurate modeling techniques. Similarly, residues falling in disallowed regions of the Ramchandran plot decreased by a good margin signifying an overall improvement in the quality of these models. Post MD Ramchandran Plot analysis has been provided in Figure 2B. Figure 4 summarizes the extent of variation in the topology of these models occurring after molecular dynamics simulations were performed.

### Comparison of structures obtained after split-domain modeling of m78 and m87 isoforms

As the SG2NA sequence contains a number of conserved domains also mentioned before, it was split into three domain regions for m78 and m87 isoforms and their structures were predicted separately using QUARK (for sequences less than 200 residues) and MUSTER as I-TASSER does not accept very short stretches of protein sequences. The three split domain regions comprised of (1) Caveolin + putative coiled-coil domain (1–140 residues) (2) Calmodulin binding domain (141–300 residues) (3) WD-40 domain. The amino acid sequences from isoforms m78 and m87 are same for caveolin and calmodulin binding domains and were submitted to QUARK. The WD-40 region is different with 301–712 residues in the m78 isoform and 301–797 residues in the m87 isoform and thus was submitted in MUSTER.

**Figure 5:**
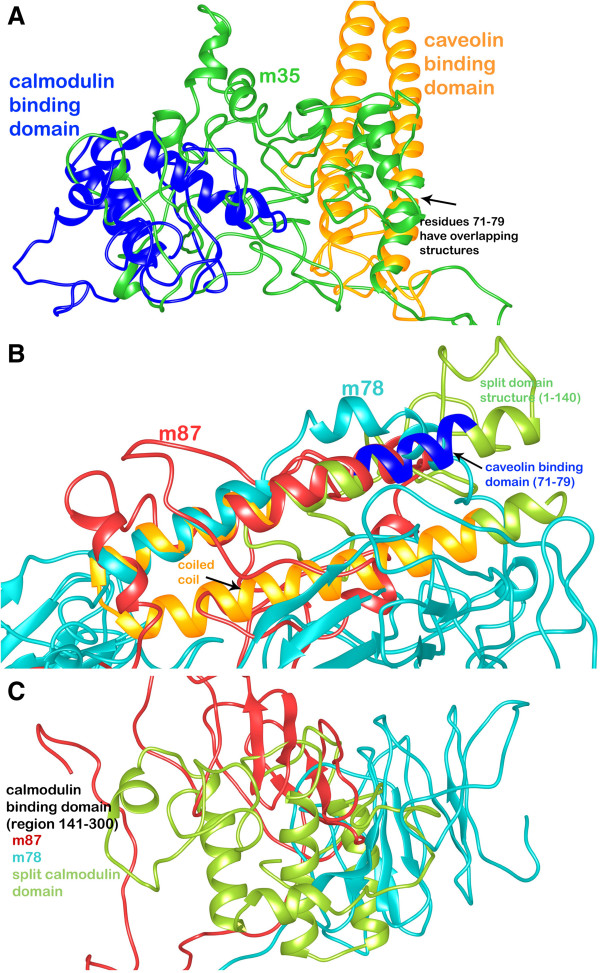
Diagram depicting split-domain comparative analysis of the caveolin-binding/coiled-coil domain model and calmodulin-binding domain model with the (A) m35, (B) m78 and (C) m87 isoforms.

**Figure 6:**
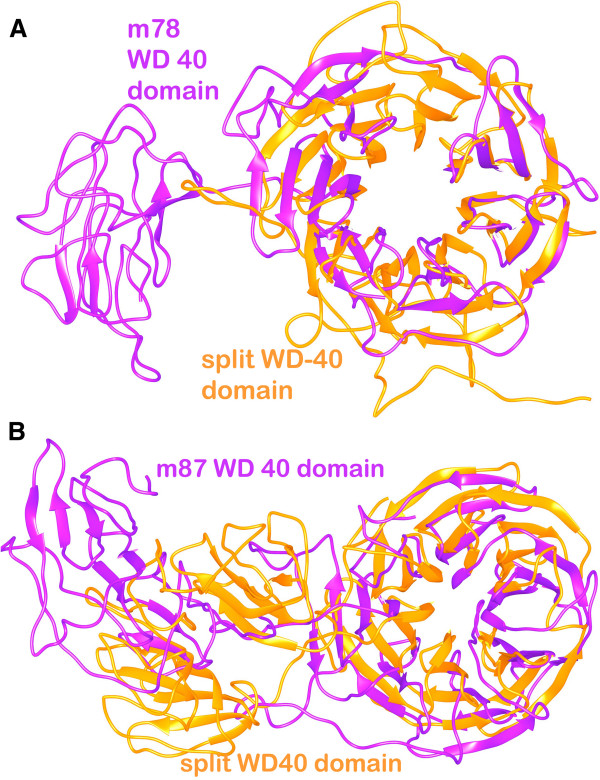
Diagram depicting split-domain comparative analysis of the WD 40 repeat domain model with the same region of (A) m78 and (B) m87 isoforms.

The separately modeled region containing Caveolin binding domain and putative coiled-coil domain (from m78 and m87 isoforms) is significantly different in the topology when compared to the complete models. As represented in Figure 5, it consists of a continuous α-helix hairpin containing caveolin binding domain (in blue) and coiled coil region (in orange). The region from 74 – 100 residues is more or less similar in all the three structures especially the caveolin binding region and thus overlaps significantly with the 3 isoforms. However, the coiled coil region predominately consists of α-helix and is completely different than all the three isoforms. The calmodulin binding region shows a completely different structure when modeled exclusively consisting majorly of helical regions whereas only loop regions are present in the complete m35 model and β-sheets are present in the same region of m78 and m87 isoforms. The possible reason for this observation can be the various stereochemical constraints under which a complete protein is modeled. In the absence of other protein domains, the algorithm just takes the single fragment into perspective and puts stereochemical constraints occurring within that part. This may not result in credible results as the true geometry is determined along with the complete protein. Finally, the WD-40 repeat domain, when compared with m78 and m87 isoforms, was found to be more or less similar exuding the same β-propeller topology except the residues from 301–500 which is topologically different even when consisting of β-sheets predominantly (Figure 6). The WD-40 repeat domain was modeled using the template based server MUSTER. The top template selected for modeling m78 isoform was the crystal structure of human TBL1XR1 WD-40 repeat protein [PDB ID: 4LG9] with a Z score of 19.63 and the top scoring template for modeling m87 isoform was highly repetitive propeller structure of WD-40 repeat protein from *Nostoc punctiforme* [PDB ID: 2YMU] with a Z score of 17.846. The Z score is given by

$$Z\;\mathrm{score}=\;\frac{\left({R^{'}}_{\mathrm{score}}-\;\left({R^{'}}_{\mathrm{score}}\right)\right)}{\sqrt{\left({R^{'}}_{\mathrm{score}}^{2}\right)-\;\left({R^{'}}_{\mathrm{score}}\right)2}}$$

Where R’_score_ is the normalized score obtained by raw alignment score (R_score_) divided by partial alignment length (L_partial_ i.e. excluding query ending gap) or R_score_ divided by full alignment length (L_full_ i.e. including query and template ending gaps). If the template chosen using L_partial_ is more similar to the query, it is chosen otherwise the template chosen by L_full_ is chosen. If the Z-score is more than 7.5, the chosen template is said to be a good template. The SG2NA proteins are known by their WD-40 repeat propeller like domain which plays a key role in its functions.

### Analysis of disorder prediction for SG2NA protein variants (35, 78 and 87 kDa)

**Figure 7:**
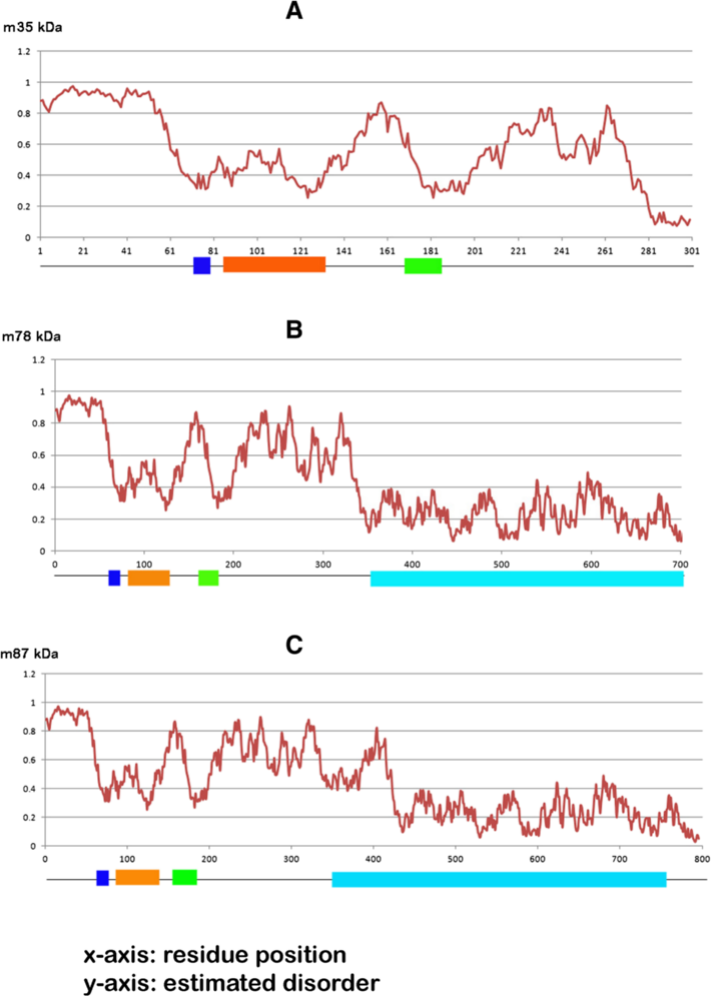
**The disorder prediction graphs of the three SG2NA protein variants (A) 35 kDa (B) 78 kDa (C) 87 kDa with respect to each residue position.** A disorder prediction probability value of >0.5 signifies that a particular residue is disordered otherwise not. The different domains have been marked along the sequence position (the Caveolin-binding domain in blue, putative coiled-coil domain in orange, Calmodulin-binding domain in green and WD-40 repeat domain in cyan).

As the SG2NA protein belongs to the three-member superfamily of WD-40 repeat containing proteins supposedly having scaffolding functions and is also known to be involved in a number of protein interactions and multimeric assembly formations. In order to exhibit such functionality with high speed of interaction but less binding strength, these proteins are ought to be disordered in their basic arrangement. Such proteins are known to have crucial functional roles in signaling pathways, cell-cycle regulation and transcription. Similarly, SG2NA is known to be involved in estrogen signaling, vesicular trafficking, spinogenesis etc. The disorder prediction results for the three protein variants have been graphically depicted in Figure 7. A disorder prediction probability value of more than 0.5 signifies disordered state of a particular amino acid residue. SG2NA variant 35 kDa, constituting just the N-terminal region was predicted to be highly disordered for most of the residue positions. This region contains the Caveolin and Calmodulin-binding domains and the putative coiled-coil structure which are expected to be highly unstable. Also, the 3D predicted model was also found to be very unstable during MD simulations further validating our disorder prediction findings. SG2NA variant 78 kDa and 87 kDa were also found to be highly disordered at the N-terminal region but became ordered for further residue positions up to the carboxylic end.

In this study, we have determined the structural specifications of the SG2NA protein that constitutes the WD-40 repeat (present at the carboxylic terminal) superfamily. The three SG2NA protein variants (35 kDa, 78kDA and 87 kDa) have been modeled using the I-TASSER server and validated using the PSVS server. The obtained models provide crucial insight into the topological specifications that are responsible for their functionality. Striatin-3 (35 kDa) is the N-terminal sequence region and thus only contains the caveolin and calmodulin-binding domains. It was found to be the most hydrophilic amongst all during initial analysis. The C-score that signifies overall quality of the model was found to be very low for this variant indicating poor model prediction. The other two variants Striatin-3 (78 and 87 kDa) have slight difference in the sequence size and thus in the overall structure. Along with a small helical caveolin-binding domain (residues 71–79) and a loop region signifying calmodulin-binding domain (residues 166–183), it contains β-sheet hairpins as the WD-40 repeat region (residues 353–753) arranged in a circular fashion in both the variants. The presence of WD-40 repeat domain in Striatin-3 is touted to be responsible for scaffolding function. A putative coiled-coil region (residues 86–132) is also present in all the three variants. The three protein models were subjected to MD simulations which resulted in relatively different folds and patterns. The SG2NA variant 35 kDa was the most variable structure amongst all during simulations. As crucial as the MD simulation can be for bringing accuracy in protein modeling process, it is accompanied with its own limitations. The present inability of computational methods for modeling native-like folds for larger proteins poses a limitation for this study as well. However, as we try to mimic biological protein folding by carrying out long MD simulations and then report the average structure of energetically stable frames, it is almost crucial to carry out MD simulation procedure. The final structures were also validated based on Ramchandran analysis with most of the residues falling in sterically allowed regions. The Ramchandran analysis scores improve significantly for MD simulated proteins as a result of removal of all unfavorable contacts in the structure. This, however, imposes another limitation to *in silico* analysis in terms of compromising the global quality of the protein model. Making as much use of available techniques, we attempted to identify the protein conformations closest to biologically relevant conformations using the available methods. Finally, owing to the multimeric organization and multi-protein interaction property of SG2NA proteins, the extent of presence of disordered regions was estimated. These results establish and propagate the structural knowledge along with certain physicochemical facts regarding SG2NA proteins that were unknown till now.

## Conclusion

SG2NA proteins are known to play vital role as diverse as cell signaling, cytokinesis, cell cycle progression, spinogenesis etc. SG2NA, owing to its disordered nature, is difficult to isolate, thus posing strong hindrances in experimental structural analysis. In view of these, computational approaches are a viable option to obtain 3D structure and to understand further the functional relationships. The analysis of the sequences revealed that 78 and 87 kDa proteins are mixture of alpha, beta and coil but containing more than 50% of random coils which make them highly flexible and nothing can be concluded about the 35 kDa protein due to low confidence score of the structure. On splitting the two isoforms m78 and m87 into three domains, it could be deduced that the caveolin binding and calmodulin binding domains are highly disordered and their structures are difficult to predict using *in silico* methods as no similar structures were found. The characteristic WD-40 repeat domain has been modeled in other organisms and bears sequence similarity with the mouse SG2NA sequence, thus, a relatively reliable structure could be obtained for this domain. Structure prediction and functional analysis of SG2NA will give an insight to the location of these proteins along with site of utilization for protein-protein interaction. This study paves way for further attempts to elucidate the experimental structures and thus validate the accuracy of the predictions. The structural knowledge of these proteins is important for deducing its mechanism of action while interacting with substrates like caveolin and calmodulin. The structural information is a pre-requisite for understanding and assigning functional role to the protein.

## Competing interests

The authors declare that they have no competing interests.

## Authors’ contributions

SS, AG and SKG designed the methods and experimental setup. SS and CT carried out the implementation of the various methods. SS, CT, AG and SKG wrote the manuscript. All authors have read and approved the final manuscript.
